# Personality vulnerability to depression, resilience, and depressive symptoms: epigenetic markers among perinatal women

**DOI:** 10.48101/ujms.v129.10603

**Published:** 2024-09-04

**Authors:** Rita T. Amiel Castro, Elena Gardini, Stavros I. Iliadis, Ulrike Ehlert, Theodora Kunovac Kallak, Alkistis Skalkidou

**Affiliations:** a Department of Clinical Psychology and Psychotherapy, University of Zurich, Institute of Psychology, Zurich, Switzerland; b Department of Women’s and Children’s Health, Uppsala University, Uppsala, Sweden; † Equal contribution

**Keywords:** Trauma, resilience, depressive symptoms, pregnancy, epigenetics

## Abstract

**Background:**

We examined differences in DNA methylation patterns in the *NR3C1* and *FKBP5* genes in relation to personality vulnerability to depression, resilience, and perinatal depressive symptoms, whilst also considering possible moderating effects of childhood traumatic events.

**Methods:**

*N* = 160 perinatal women were assessed at late pregnancy and 1 year postpartum for personality vulnerability to depression, resilience, depressive symptoms, and childhood traumatic events with self-reported questionnaires. *NR3C1* and *FKBP5* methylation markers were analyzed via sodium bisulfite sequencing. Associations of methylation markers with the above mentioned variables were tested using multivariable regressions.

**Results:**

*NR3C1* methylation at CpGs 1, 4 and average methylation sites were negatively associated with resilience; *NR3C1* methylation at CpG 2 was positively associated with postpartum depressive symptoms; methylation at CpG 4 was positively associated with prenatal depressive symptoms. The interaction between current distress due to interpersonal traumatic events and *NR3C1* CpG sites in relation to personality vulnerability was significant on CpG sites 3 and 4, whereas the interaction between current distress due to total traumatic events and *NR3C1* in relation to personality vulnerability was significant on CpG site 2. *FKBP5* showed no significant associations with the outcomes.

**Conclusions:**

This study identified associations between NR3C1 methylation and resilience as well as perinatal depressive symptoms. Interestingly, an interaction between early trauma and personality vulnerability was noted. Our findings on these specific DNA methylation markers may, if replicated and integrated into risk prediction models, contribute to early diagnosis of mothers at risk, targeted health promotion, and early interventions.

## Introduction

Childhood traumatic or stressful events constitute a serious risk factor for long-term biological, developmental, and psychological disturbances (1–3). The implications of early traumatic experiences include a higher risk of psychiatric disorders from childhood to adulthood, such as depression, anxiety, somatization, and posttraumatic stress disorder (4). Moreover, in women, the experience of early life stressful events early in life increases the risk of developing mental disorders during pregnancy and postpartum (4).

Interestingly, several studies highlight the fact that many individuals who suffered childhood trauma will not develop later psychopathology (5–7). For instance, one study found that 48% of children who experienced abuse and neglect did not go on to meet any diagnostic criteria for adult psychiatric disorders, and 38% did not meet criteria for substance abuse. Individuals who develop adaptive responses to risk are normally referred to as resilient (8). Understanding the processes and factors involved in positive adaptation to adversity has been shown to be vital to promote psychological resilience (9). Resilience is dynamic and can be defined as successful adaptation after experiencing adversity, trauma, tragedy, threats of harm, or high levels of stress (10). Among children who suffered childhood trauma such as abuse or neglect, 22% were classified as resilient due to their successful functioning in a broad range of psychosocial domains in adulthood (8). Resilient functioning appears to arise from an interaction between heritable factors, personal characteristics, and experiences over time (11). Emotionally responsive parenting, active coping, adequate emotion regulation, and supportive relationships are likely to play an important role in this regard (9).

Similar to environmental variables, epigenetics also seems to influence a person’s vulnerability or resilience to mental disorders (12). Childhood traumatic experiences seem to for can negatively alter the hypothalamic-pituitary-adrenal (HPA) axis functioning, which is the main system facilitating stress regulation (13, 14). Over the past decade, research findings have pointed to epigenetic mechanisms as a process by which environmental factors may alter gene expression related to the control of the HPA axis (15).

Studies have demonstrated associations between epigenetic changes in the nuclear receptor subfamily 3 group C member 1 (*NR3C1*) gene with adverse life events and psychopathology. *NR3C1* encodes the glucocorticoid receptor (GR), which seems to have its signalling disrupted in anxiety and depression disorders, particularly in the context of early traumatic events (16). Numerous genetic variants have been associated with functional changes of the GR (17). For instance, the FK506 binding protein 5 (*FKBP5*), an important regulator of the GR complex, alters the GR by reducing the ligand binding and preventing the transference of the GR complex to the nucleus (18). Epigenetic modifications in the *FKBP5* gene have been shown to be associated with early trauma, posttraumatic stress disorder, as well as depression later in life (18, 19).

While a combination of heritable factors, personal characteristics, and experiences is suggested to be relevant for resilience, less is known about the reasons for its variability among pregnant and postpartum women with a history of childhood trauma (20). Childhood trauma negatively impacts adaptation to motherhood, specifically the ability to be a sensitive and responsive parent (21). Moreover, mother–baby bonding is especially difficult for women who experienced childhood traumatic events (22). Few studies have investigated past traumatic experiences and resilience in pregnant women. So far, research has attempted to highlight several pathways that may account for resilience following early adverse events, but important gaps still remain (23, 24). Epigenetic variations in the *NR3C1* and the *FKBP5* genes as a result of childhood trauma have been implicated as possible mechanisms. There is also insufficient evidence regarding potential independent effects of, or an interaction between, DNA methylation and early trauma in predicting perinatal depressive symptoms and a higher vulnerability to the development thereof. Moreover, it remains unclear whether there is a moderating effect of DNA methylation in the association between early trauma and perinatal depression. A greater understanding of these issues might lead to early identification and appropriate treatment for individuals at risk.

Therefore, the present study aims to (1) examine individual differences in DNA methylation patterns within the GR receptor *NR3C1* and the *FKBP5* genes in relation to personality vulnerability to depression, resilience, and perinatal depressive symptoms, whilst also controlling for early life traumatic events, and (2) assess the possible moderating effects of traumatic events on the association between DNA methylation and the aforementioned outcomes. We hypothesize that early trauma will enhance the association between DNA methylation and the outcomes of interest. The consideration of epigenetic susceptibility factors together with psychological markers is not only theoretically important, insofar as models of resilience may improve our understanding of the mechanisms linking trauma, resilience, and psychopathology, but is also clinically relevant for future risk identification programs and interventions.

## Material and methods

### Study population

Participants were part of a longitudinal study examining women’s perinatal psychological wellbeing, and were recruited between 2009 and 2019 in Uppsala County, Sweden. Pregnant women attending the routine ultrasound examination at the Uppsala University Hospital in pregnancy weeks 17–19 received written information and were invited to take part in the BASIC (Biology, Affect, Stress, Imaging and Cognition) study (25). Inclusion criteria were: (1) age over 18 years, (2) ability to communicate in Swedish, (3) a normal pregnancy as diagnosed by routine ultrasound, (4) non-confidential personal data, and (5) no known blood-borne disease. Following recruitment, eligible women read the study information and those who agreed to participate signed a written consent form. Informed consent was obtained from participants prior to study commencement.

Participants of the current sub-study provided data at different time points from pregnancy week 17–12 months postpartum (25). Consenting women provided a sample of venous blood at delivery (96%) or in late pregnancy (4%). Complete data was available for *N* = 303 women. Ethical approval was obtained from the Regional Ethical Review board (DNR 2009/171, with amendments) in Uppsala, Sweden, before commencement of the study. The study protocol was performed in accordance with relevant ethical guidelines and regulations and followed the Declaration of Helsinki.

### Measures

#### The Vulnerability Personality Style Questionnaire

The Vulnerability Personality Style Questionnaire (VPSQ) (26) is a self-report questionnaire comprising nine items that are rated on a 5-point Likert scale (1 = not correct at all, 5 = exactly right). It was developed to evaluate personality dimensions associated with an increased risk of developing postpartum depression. Items are summed to produce a total score ranging from 9 to 45, with higher scores indicating increased vulnerability. The personality dimensions assessed by the VPSQ are: nervy, timidity, sensitivity, worrier, organized, obsessive, expressive, volatility, and coping. Higher scores indicate a greater risk of developing postpartum depression. The VPSQ was assessed at 12 months postpartum.

#### Resilience Scale 14

The Resilience Scale 14 (RS-14) (27, 28) is a self-report scale consisting of 14 items that are rated on a 7-point Likert scale ranging from 1 = strongly disagree to 7 = strongly agree. Example items are ‘I usually manage one way or another’, ‘I am determined’, and ‘I can get through difficult times because I’ve experienced difficulty before’. Total scores range from 14 to 98, with scores below 65 indicating low resilience, scores between 65 and 81 indicating moderate resilience, and scores above 81 indicating high resilience. Resilience was assessed at pregnancy week 32.

#### Edinburgh Postnatal Depression Scale

The Edinburgh Postnatal Depression Scale (EPDS) (29, 30) is a 10-item self-report screening tool developed to specifically assess depressive symptoms in the postpartum period. Items are rated referring to the past week on a 4-point scale (0–3), with the total score ranging from 0 to 30. The instrument has shown good psychometric properties both in pregnancy and in the postpartum period. We refer to depressive symptoms as the total scores calculated from the questions included in this instrument (used as a continuous variable). The EPDS was assessed at pregnancy week 32 and at 6 weeks postpartum.

#### Lifetime Incidence of Traumatic Events

The Lifetime Incidence of Traumatic Events (LITE) (31, 32) is a 16-item checklist used to assess a history of childhood traumatic and adverse events. For each endorsed event, the instrument captures the respondent’s age at the time of exposure, the frequency of occurrence, and the current distress level pertaining to the respective event. The items ask about the occurrence of accidents, threats, sexual assaults, natural disasters, and other potentially upsetting events (e.g. Have you been in a car accident? If yes: how many times?; how old were you?; how much did it upset you then?; how much does it bother you now?). The level of distress is measured on a 3-point Likert scale (0 = not at all, 1 = slightly, 2 = very much), with higher scores indicating a higher distress level. In the present study, the total number of traumatic events was used as a continuous measure. Only events occurring before the age of 18 were considered. Moreover, the distress level and the number of traumatic events were also categorized within two subscales: interpersonal events (e.g. Were you forced into sexual acts?) and non-interpersonal events (e.g. Have you seen someone get hurt?), as there is considerable evidence that specific trauma types have differential impacts on mental health (33, 34). Total and interpersonal childhood traumatic events were analyzed separately. Childhood traumatic events were retrospectively assessed at 12 months postpartum.

#### Blood sampling

Venous blood samples were collected in late pregnancy (35–39 weeks gestation) or at the time of delivery in EDTA-containing tubes separating plasma and buffy coat. Samples were stored at −70°C until further analysis.

#### DNA methylation

**Figure 1:**
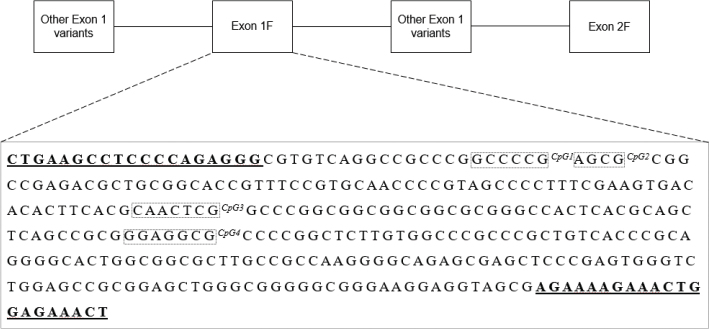
The upper panel represents the diagram of the first exon in the *NR3C1* promoter region. The lower panel depicts the *NR3C1* exon 1F sequence, chr5 (hg19): 142,783,586–142,783,903 located in the 5′untranslated region of the *NR3C1*. CpG 1–4 correspond to CpG sites 37, 36, 26, and 16 described by Palma-Gudiel (37). Underlined sequences correspond to the primer’s positions.

**Figure 2:**
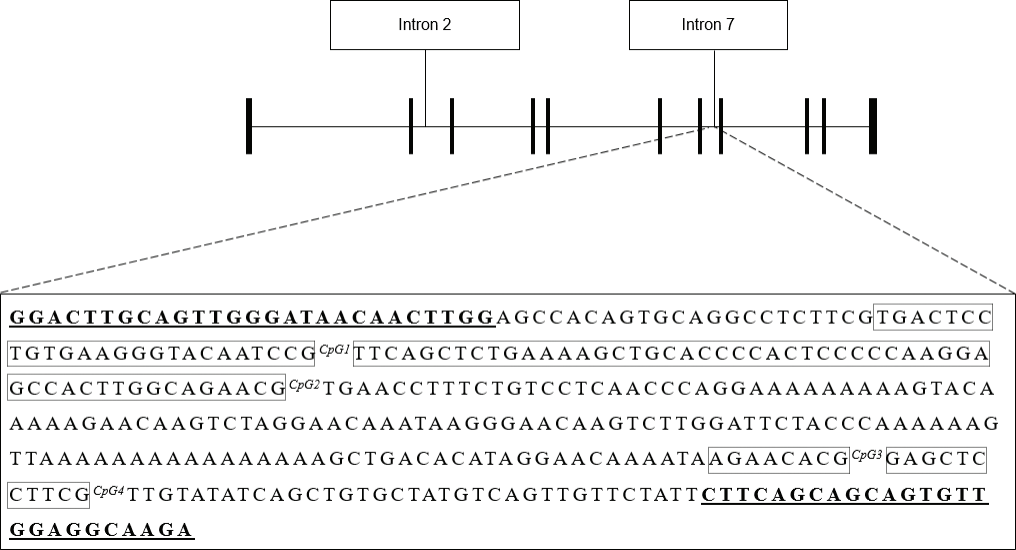
The upper panel represents the *FKBP5* locus including intron 7 GR. Black bars represent the 11 exons. The lower panel depicts the *FKBP5* intron 7 sequence, chr6 (hg19): 35558441–35558783. The CpG sites are represented in bold boxes as described by Klengel et al. (18) In Klengel et al. and Yehuda et al. (10), our CpG 1 corresponds to their CpG 3, our CpG 2 corresponds to their CpG 4, our CpG 3 corresponds to their CpG 5, and our CpG 4 corresponds to their CpG 6. Underlined sequences correspond to the primer’s positions.

DNA was extracted from buffy coat using the silica-based Kleargene™ XL nucleic acid extraction kit (^®^LGC, UK) or the Chemagen kit based on magnetic bead separation on a Chemagic Star^®^ Robot (Hamilton Robotics, Reno, NV, USA). Sodium bisulfite conversion was conducted using the EZ DNA methylation kit (500 ng of DNA; Zymo Research, Irvine, CA, USA). The amplification of the *NR3C1* and *FKBP5* target sequence was performed following bisulfite primers. Bisulfite primers included universal primer sequences CS1/CS2 on the 5*′* ends (Fluidigm, San Francisco, CA, USA). Cycling conditions used in the analyses were: 95°C for 3 min, then 40× (98°C for 20 s, 60°C for 15 s, 72°C for 15 s), and final elongation step at 72°C for 45 s. Next, amplicons were purified using E-gel size selection (Thermo Fisher Scientific, Waltham, MA, USA). Samples were then indexed with unique single barcodes (Fluidigm, San Francisco, CA, USA) through a second polymerase chain reaction (PCR) (95°C, 3 min, then 10× (98°C, 20 s; 60°C, 15 s; 72°C, 15 s), and final elongation at 72°C for 45 s). Indexed amplicons were pooled and submitted to a final purification to remove dimers and amplification artifacts. The pooled samples were then diluted to a concentration of 2 nM and sequenced on an Illumina Miseq using the v3 kit (Illumina, San Diego, CA, USA). After DNA sequencing, we used the Trimmomatic v0.35 software (http://www.usadellab.org/cms/index.php?page=trimmomatic) to identify and remove low-quality products (35). To extract the counts of methylated (cytosines) and unmethylated (thymine), bases, we used the Bismark program (v0.19.0). After summing up methylated and unmethylated counts, we only kept samples showing a coverage of at least 100× as recommended by Chen and colleagues (36). *N* = 143 samples did not reach this threshold and were thus excluded. The remaining samples (*n* = 160) showed a coverage ranging from 119^x^ to 135^x^ (*NR3C1*; Mean = 127.8 *SD* = 125.3) and from 894.5^x^ to 10238.5^x^ (*FKBP5*; Mean = 6384.9; *SD* = 5205.4). We investigated the methylation pattern of the *NR3C1* exon 1F region, specifically examining four CpG sites in exon 1F (Figure 1) located within the 35th through 37th and the 1^st^ through 29^th^ CpG sites described by Palma-Gudiel et al. (37). The methylation patterns of four CpGs in intron 7 from *FKBP5* were also studied based on findings from Klengel and colleagues (18) (Figure 2). The individual methylation percentages at the CpG sites and their average values were used in the analyses.

### Statistical analyses

Our analyses focused on targeted genes (*NR3C1* and *FKBP5*) with gene selection based on previous literature on this topic. We generated our hypotheses and targeted sample size considering these genes.

Statistical analyses were performed using the IBM Statistical Package for the Social Sciences (SPSS Version 24 for Windows), whereas a power analysis was conducted with G* Power version 3.1.9.7. Since all variables were skewed, we applied log transformation using the formula log10 (value + 1). We used non-parametric tests with non-transformed values (e.g. Spearman correlations). In addition, the log-transformed dependent variables were used in the regression models, in which the non-standardized residuals were checked for normality. Spearman correlations were conducted between personality vulnerability to depression, resilience, depressive symptoms, covariates, *NR3C1* and *FKBP5* methylation percentage, and traumatic events (total and interpersonal). Covariates were specified *a priori* based on previously published determinants of maternal emotional state in pregnancy, and included the variables listed in Supplementary Table 1. All analyses were adjusted for these covariates. Although we did not have sufficient variability within the sample to control for smoking (99.5% were non-smokers), we repeated our analyses excluding the two participants who smoked. The associations of *NR3C1* and *FKBP5* methylation values with outcomes (personality vulnerability to depression, resilience, and depressive symptoms) were tested using Mann–Whitney U tests. For the transformation of these variables into binary ones, we used the median score for each variable of interest in our sample in order to create the two categories (high vs. low), for example the values above and below that value respectively.

Multiple linear regression analysis was used to examine associations between *NR3C1* and *FKBP5* methylation (average and individual) and outcomes. Additionally, we also included traumatic events and covariates, yielding the following model: outcome ~ methylation + traumatic events + covariates. In order to understand whether the number of traumatic events (total and interpersonal; binary variable; 0 = 0–1 event and 1 = >1 event) and current distress level for each endorsed event (total and interpersonal; binary variable; 0 = no distress, 1 = moderate/severe distress) moderate the association between predictors and outcomes, we estimated interaction terms between each *NR3C1* and *FKBP5* CpG site and the two aforementioned variables. All tests were conducted on four CpG sites and the mean methylation level across these sites. A Bonferroni-corrected significance level of *p* = 0.01 was used to adjust for multiple testing in all our tests. Multicollinearity was assessed using the variance inflation factor (VIF), and no issues were found (e.g. all VIFs were < 2). To facilitate the interpretation of the regression coefficients, we rescaled the predictors and outcomes by multiplying them by 10.

## Results

Of the 303 participants with relevant data, *N* = 160 had DNA samples showing a coverage of at least 100×^36^ – the recommended threshold of acceptance – and were included in the analyses. *A posteriori* power analysis indicated that with *N* = 160, we are able to achieve an 80% power for detecting a small to medium effect (38) (*f* ^2^ = 0.11), at a significance criterion of α = 0.01, for the tests conducted.

Characteristics of the sample are presented in Table 1. Participants were on average 31 years old (SD = 3.84), and 64.5% had pregnancy complications. A total of 34.4% of our sample had suffered a childhood trauma, 22.5% had experienced one or more interpersonal traumatic events, and 13.7% reported slight or high current interpersonal distress. On average, participants reported having experienced one childhood traumatic event (SD = 1.85).

**Table 1:** Sample characteristics.

Variables	*N*	Missing	Median	IQR	%
Maternal age	160	0	31	7.00	
Previous psychological treatment or medical history of depression (Y)	160	0			43.8
Pregnancy complications (Y)	160	0			62.5
Primiparous	157	3			61.1
BMI (kg/m^2^)	160	0	22.38	4.21	
Depressive symptoms (EPDS)	160	0	5	6.00	
Traumatic events – number of events (LITE)	160	0	1	2.00	
Traumatic events – current distress level (LITE)	160	0	0.4	0.00	
Resilience (RS-14)	160	0	81	14.00	
Personality vulnerability to depression (VPSQ)	160	0	21	9.00	
Epigenetic Data					
NR3C1					
CpG 1	160	0	0.83	0.51	
CpG 2	160	0	0.77	0.72	
CpG 3	160	0	3.06	0.85	
CpG 4	160	0	3.30	0.43	
Mean methylation level	160		1.99	0.61	
FKBP5	160				
CpG 1	160	0	97.66	3.30	
CpG 2	160	0	76.57	9.67	
CpG 3	160	0	63.14	12.03	
CpG 4	160	0	58.81	12.06	
Mean methylation level	160	0	73.93	8.23	

Note: Y = Yes. Pregnancy complications include gestational diabetes, preeclampsia, anemia, IQR = Interquartile range; BMI = body mass index; EPDS = Edinburgh Postnatal Depression Scale; LITE = Lifetime Incidence of Traumatic Events; VPSQ = Vulnerability Personality Style Questionnaire; RS-14 = Resilience Scale 14.

### Bivariate associations between NR3C1 and FKBP5 methylation and outcomes

DNA methylation of *NR3C1* and *FKBP5* was not significantly correlated with personality vulnerability to depression, resilience levels, or with perinatal depressive symptoms. All *FKBP5* CpG units, as well as average CpG, were however negatively correlated with previous psychological treatment or history of depression (ranging from *r_s_* = –0.19 to –0.25, *p* < 0.01).

A Mann–Whitney U test indicated that women with lower personality vulnerability to depression had higher *NR3C1* methylation at CpG site 2 as compared to those with higher personality vulnerability (*p* < 0.01). There were no other group differences in *NR3C1* CpGs and *FKBP5* methylation in relation to the outcomes (resilience, depressive symptoms, and personality vulnerability to depression).

### Correlations between traumatic events and personality vulnerability to depression, resilience, depressive symptoms, and DNA methylation

The number of *total* traumatic events, assessed at 1 year postpartum, was positively associated with depressive symptoms at 32 weeks gestation (*r_s_* = 0.17, *p* < 0.01). The number of *interpersonal* traumatic events was positively correlated only with personality vulnerability to depression (*r_s_* = 0.19, *p* < 0.01). Neither total traumatic events nor interpersonal traumatic events, assessed at 1 year postpartum, were significantly correlated with DNA methylation of either *NR3C1* or *FKBP5*, or resilience – assessed at 32 weeks gestation. Current distress level due to a *total* traumatic event was positively associated with personality vulnerability (*r_s_* = 0.22, *p* < 0.01) and with depressive symptoms at 32 weeks gestation (*r_s_* = 0.30, *p* < 0.01) and at 6 weeks postpartum (*r_s_* = 0.39, *p* < 0.01). Current distress level due to *total* and an *interpersonal* traumatic event was positively correlated with personality vulnerability (*r_s_* = 0.23, *p* < 0.01), and with depressive symptoms at 32 weeks gestation (*r_s_* = 0.23, *p* < 0.01) and at 6 weeks postpartum (*r_s_* = 0.23, *p* < 0.01). Again, current distress level due to a *total* or *interpersonal* traumatic event was not significantly correlated with DNA methylation in both *NR3C1* and *FKBP5* genes or with resilience.

### Adjusted multivariable regressions between NR3C1 and FKBP5 methylation and outcomes

*NR3C1* methylation at CpG site 1, CpG site 4, as well as average methylation percentage, were negatively associated with resilience level after controlling for covariates such as maternal age, pregnancy complications, parity, history of depression, and pre-pregnancy BMI. This means that a 10% lower methylation percentage is associated with a resilience that is higher by 10 units (see Table 2). *NR3C1* methylation at CpG site 4 (*B* = 0.05, CI95% 0.01 – 0.1) was positively associated with depressive symptoms during pregnancy after controlling for covariates. Similarly, *NR3C1* CpG 2 was positively associated with postpartum depressive symptoms (see Table 3). No other associations were found with *NR3C1*. All CpG individual and average sites within *FKBP5* showed no significant associations with personality vulnerability to depression, resilience, or perinatal depressive symptoms. Running the analyses separately for total number of traumatic events, current distress level as well as for number of interpersonal traumatic events and current distress level regarding interpersonal events yielded mostly non-significant findings (see Supplementary Tables 2–8).

**Table 2:** Adjusted multivariable regressions between *NR3C1* CpG sites and mean methylation in relation to resilience.

Predictors^a^	*B*	CI (95%)	*p*
Medical history of depression	-2.3	-2.3 – 0.2	0.01
Interpersonal events	-1.0	-1.0 – 0.1	n.s.
CpG 1	-2.9	-3.5 – 0.1	0.00
Medical history of depression	-2.2	-2.3 – 0.1	0.02
Interpersonal events	-1.1	-1.3 – 0.1	n.s.
CpG 2	-2.1	-2.3 – 0.0	n.s.
Medical history of depression	-2.1	-2.3 – 0.1	n.s.
Interpersonal events	-1.0	-1.3 – 0.1	n.s.
CpG 3	-2.7	-3.1 – 0.0	n.s.
Medical history of depression	-0.1	-1.3 – 0.2	0.01
Interpersonal events	-0.9	-0.9 – 0.1	n.s.
CpG 4	-3.1	-3.1 – 0.0	0.00
Medical history of depression	-2.2	-2.3 – 0.2	0.01
Interpersonal events	-1.0	-1.3 – 0.1	n.s.
Mean methylation level	-2.9	-3.2 – 0.1	0.00

Note: BMI = body mass index,

a All models were also adjusted for maternal age, pregnancy complications, parity, and BMI.

**Table 3:** Adjusted multivariable regressions between *NR3C1* CpG sites and mean methylation in relation to depressive symptoms postpartum.

Predictors^a^	*B*	CI (95%)	*p*
Medical history of depression	4.1	1.6 – 4.2	0.00
Interpersonal events	0.4	-0.3 – 0.5	n.s.
CpG 1	1.8	0.0 – 1.9	n.s.
Medical history of depression	4.2	1.7 – 4.3	0.00
Interpersonal events	0.4	-0.3 – 0.5	n.s.
CpG 2	2.1	0.1 – 2.7	0.01
Medical history of depression	4.0	1.6 – 4.0	0.00
Interpersonal events	0.4	-0.3 – 0.5	n.s.
CpG 3	1.7	0.0 – 2.2	n.s.
Medical history of depression	4.0	1.6 – 4.0	0.00
Interpersonal events	0.3	-0.3 – 0.5	n.s.
CpG 4	0.05	0.01 – 0.09	n.s.
Medical history of depression	4.1	1.6 – 4.2	0.00
Interpersonal events	0.4	-0.3 – 0.5	n.s.
Mean methylation level	1.9	0.0 – 2.3	n.s.

Note: BMI = body mass index,

a All models were also adjusted for maternal age, pregnancy complications, parity, and BMI.

Results from the interaction between current distress level due to each interpersonal traumatic event and each *NR3C1* CpG site in relation to resilience revealed no statistically significant findings. In addition, the interaction term relating to current distress level due to each interpersonal traumatic event and each *NR3C1* CpG site in relation to prenatal and postpartum depressive symptoms was not significant (*p* > 0.01). Finally, the interaction term relating to current distress level due to each interpersonal traumatic event and each *NR3C1* CpG site in relation to personality vulnerability to depression was significant on CpG 3 and 4 (*B* = 0.50, CI95% 0.01–0.60, *B* = 0.70, CI95% 0.16–1.24, respectively). Moreover, the interaction term between current distress level due to total traumatic event and each *NR3C1* CpG site in relation to personality vulnerability to depression was significant on CpG 2 (*B* = 0.07, CI95% 0.01–0.13). We also created and analyzed the terms between current distress level due to each interpersonal traumatic event and all *FKBP5* sites; no significant results in relation to any outcome were found.

Similarly, interaction terms between *NR3C1* methylation sites and total traumatic events, *FKBP5* methylation sites and total traumatic events, *NR3C1* methylation sites and interpersonal traumatic events and *FKBP5* methylation sites and interpersonal traumatic events in relation to resilience, perinatal depressive symptoms, and personality vulnerability to depression did not reveal any statistically significant findings. All analyses were repeated after excluding the two individuals who smoked, and the results remained unchanged.

## Discussion

We examined individual differences in the DNA methylation patterns within the GR receptor *NR3C1* and the *FKBP5* gene in order to characterize epigenetic markers of personality vulnerability to depression, resilience, and perinatal depressive symptoms. Moreover, we tested possible moderating effects of early trauma on the aforementioned associations. We found that high resilience was associated with decreased *NR3C1* methylation, whereas depressive symptoms in pregnancy and postpartum were associated with increased *NR3C1* methylation. Furthermore, we detected an interaction effect between current distress level due to interpersonal traumatic events in childhood and *NR3C1* CpG sites 3 and 4, in relation to personality vulnerability to depression.

Stress accumulation over the lifespan can contribute to biological vulnerability and affect health outcomes (39). DNA methylation is influenced by various environmental factors, with epigenetic changes at *NR3C1* being strongly linked to exposure to early life stress (40). The *NR3C1* genomic locus has been well-investigated (37). Methylation in this promoter region has been systematically associated with psychopathology and early adversity (37). The CpG 1 investigated here has been previously linked to maternal anxiety and depressive symptoms in pregnancy in newborns with elevated methylation (41), as well as to early parental loss and early maltreatment in adults with increased methylation (42). Moreover, it has been also associated with hypomethylation in patients with posttraumatic stress disorder (43) and in newborns exposed to maternal smoking or psychopathology during pregnancy (44). Methylation at this specific site has been found to be predictive of HPA reactivity and of cortisol response (41, 42). The CpG 2 has also been consistently associated with maternal depression, anxiety, and fear of childbirth in the case of newborns presenting with hypermethylation (44). On the other hand, evidence on the effects and function of CpG 3 and CpG 4 is limited, with the available studies suggesting an association between increased methylation and early life stress (44, 45). The latter concurs with our moderation finding that increased current distress level due to interpersonal traumatic events in childhood and increased methylation on *NR3C1* CpG sites 3 and 4 heighten personality vulnerability to depression.

We observed decreased methylation of the *NR3C1* individual and average CpG sites among those with high resilience levels. Our findings are in line with the only available study examining the role of *NR3C1* methylation in resilience among a student population (46). Specifically, Miller and colleagues (46) reported that methylation of *NR3C1* sites predicted both reduced and heightened resilience, with no observable patterns justifying the links between opposing resilience levels and methylation regions. There is limited literature on studies involving DNA methylation and positive responses to trauma (47). Our findings suggest a theoretically plausible positive biological effect of resilience (47) on early traumatic experiences, although it is notable that we assessed resilience many years after the traumatic experiences via self-report. It is suggested that resilience is affected by epigenetic signatures present from conception, by early life environmental impacts and by the genetic moderations of the environment on the epigenome (48). The lack of literature in this area, and the potential for an epigenetic influence on resilience, highlight the need for further research. Our findings might thus contribute in better understanding the biological mechanisms in shaping resilience after early traumatic experiences.

Moreover, the present findings suggest that increased methylation of the *NR3C1* is associated with a higher level of depressive symptoms in pregnancy and postpartum. This is in line with the results of clinical studies involving non-perinatal populations (49). By encoding the human GR protein in which cortisol and other glucocorticoids bind, *NR3C1* exerts a regulatory impact on HPA axis functioning, which is in turn involved in stress reactions (50). However, the opposite findings, as well as CpG site-specific findings, have also been reported. Recent reviews (40, 47) on *NR3C1* DNA methylation highlighted the complexity of the associations pertaining to this epigenetic system in relation to depression, suggesting a potential inverted U-shaped relationship similar to that described for peripheral cortisol and depression (51). These findings emphasize the need for continued research on the directionality of epigenetic modification and depression. Notably, the majority of studies investigating *NR3C1* methylation and perinatal mental health focused on understanding the effects of maternal mental health on newborn methylation (52). While this is important, as there may be direct implications for the developing child, early life trauma exposure in the mothers can also result in long-lasting changes in maternal DNA methylation, which may lead to psychiatric disorders in adulthood, including the pregnancy and postpartum phases (53).

The *FKBP5* is an important regulator of the GR, influencing GR sensitivity and stress response regulation. It has been shown that CpG sites in intron 7 are differentially methylated when exposed to childhood abuse in the presence of the *FKBP5* rs1360780 risk allele (18). This can be explained by the fact that intron 7 CpG sites are demethylated by glucocorticoids – as they are located close to functional consensus glucocorticoid response elements – especially during important developmental phases (18). Reduced methylation of our CpGs 1, 2, and 3 has been shown to be associated with early abuse in the presence of the *FKBP5* risk allele (18), whereas at CpGs 1, 2, 3, and 4, demethylation was achieved by glucocorticoid administration (18). Contrary to previous studies (46), we found no associations between *FKBP5* and resilience levels. It is unclear why we did not find any associations with this specific stress-related gene, although potential reasons may lie in the specificity of the targeted methylation analyses. Further, our findings did not support a consistent association between *FKBP5* methylation and personality vulnerability to depression or with perinatal depressive symptoms. Therefore, the present results are in contrast to the literature pointing to *FKBP5* epigenetic modifications in traumatized and depressed individuals (49). The fact that previous studies did not use exactly the same CpGs and transcriptional binding sites as in the present study, together with different pathways used for the data approach, may preclude comparison of our results with previous findings. A further reason might be that we analyzed a homogeneous sample of mostly Scandinavian origin, comprising highly educated women with low levels of trauma, who were mostly healthy or had mild self-reported depressive symptoms. Further, perinatal depressive symptoms might have another pathophysiology than depression in other life phases, and this needs to be also considered.

This study is limited by the homogeneous sample size that restricts the generalizability of the findings. Importantly, we adjusted our analyses for multiple testing to correct for occurrence of false positives. This means that our findings reflect reliable associations. Although our effect sizes were small, they could provide further insights in the complex pathophysiology of early trauma and resilience. Moreover, we analyzed methylation patterns in peripheral tissues, which cannot be assumed to reflect DNA methylation in relevant central nervous system regions. However, it has been suggested that methylation in peripheral blood and brain is highly correlated (54). Given that we did not conduct an epigenome wide methylation analysis, we could not estimate cell type-specific methylation. Although post-traumatic stress disorder was previously associated with both genes studied here, we did not assess it since this was not our study aim. We also did not measure depressive symptoms concurrently with the VPSQ, which could be seen as a limitation, as we cannot control for the possibility of a state effect. Finally, we cannot rule out a report and/or memory bias in the self-report measures, which might have led to misclassification and dilution of associations. These limitations are offset to a considerable degree by several strengths of the study, such as addressing the research gap on biological predictors of resilience and perinatal psychopathology, the use of a highly accurate and sensitive DNA methylation method, and the candidate gene design.

In sum, our results suggest lower *NR3C1* methylation among those with high resilience levels, whereas higher *NR3C1* methylation was associated with perinatal depressive symptoms. Self-reported early trauma did not enhance the associations between DNA methylation and the outcomes. Nevertheless, there were interaction effects between *current distress* levels due to childhood interpersonal traumatic events and total events and *NR3C1* methylation in relation to personality vulnerability to depression. Our study contributes to the limited field of epigenetic aspects of childhood trauma literature, while moving the field forward by examining epigenetic modifications on post-trauma responses in perinatal women. DNA methylation markers found in the current study might contribute, together with other variables, to early diagnosis of mothers at risk, targeted health promotion, and prevention or timely treatment. Future longitudinal studies investigating epigenetic changes from pre to post pregnancy in women who previously experienced early trauma, and how these might influence the effects of early trauma are warranted. Further investigations using a broadened research approach and targeting other relevant genes and pathways should increase our understanding of epigenetic mechanisms of resilience and psychopathology.

## Supplementary Material



## Data Availability

Currently, the General Data Protection Regulation (GDPR), hinder data deposition of human genetic data. The data and syntaxes used in this study are available from the authors upon reasonable request and with permission from the BASIC study cohort.
