# Supplementary material for: Personality vulnerability to depression, resilience, and depressive symptoms: epigenetic markers among perinatal women
Source: Ups J Med Sci. 2024 Sep 4;129:10.48101/ujms.v129.10603. doi: 10.48101/ujms.v129.10603 (PMC11385460; doi:10.48101/ujms.v129.10603)
Supplement: Supplementary file 1 [file UJMS-129-10603-s001.pdf]

Supplementary Table 1. Covariates overview.

**Variable**	**Variable Type**
Maternal age	Continuous variable
Pregnancy complications (i.e. gestational diabetes, preeclampsia, and anemia)	Binary variables coded as yes vs. no
Parity	Continuous variable
Previous psychological treatment or history of depression	Binary variable, yes vs. no
Pre-pregnancy body mass index (BMI)*	Continuous variable

Note: *BMI was included as a covariate due to its influence on DNA methylation (Dick et al., 2014).

Supplementary Table 2. Linear regression analyses between *NR3C1* CpG sites and mean methylation in relation to resilience adjusted for current distress level endorsed by the traumatic event.

Predictors	B	CI (95%)	p
Maternal Age	0.01	-0.06, 0.09	n.s.
Medical history of depression	-0.68	-1.28, -0.08	n.s.
Pregnancy complications	-0.56	-1.18, 0.06	n.s.
Parity	0.03	-0.40, 0.46	n.s.
BMI	0.04	-0.03, 0.11	n.s.
Current distress level	-0.14	-0.34, 0.06	n.s.
CpG 1	-0.06	-0.14, 0.03	n.s.
Maternal Age	0.01	-0.06, 0.09	n.s.
Medical history of depression	-0.74	-1.35, 0.13	n.s.
Pregnancy complications	-0.55	-1.17, 0.07	n.s.
Parity	0.04	-0.39, 0.47	n.s.
BMI	0.04	-0.03, 0.11	n.s.
Current distress level	-0.14	-0.34, 0.06	n.s.
CpG 2	-0.08	-0.20, 0.04	n.s.
Maternal Age	0.01	-0.06, 0.09	n.s.
Medical history of depression	-0.70	-1.30, -0.10	n.s.
Pregnancy complications	-0.55	-1.17, 0.07	n.s.
Parity	0.05	-0.38, 0.48	n.s.
BMI	0.04	-0.03, 0.11	n.s.
Current distress level	-0.14	-0.34, 0.06	n.s.
CpG 3	-0.02	-0.04, 0.01	n.s.
Maternal Age	0.10	-0.06, 0.09	n.s.
Medical history of depression	-0.70	-1.30, -0.10	n.s.
Pregnancy complications	-0.56	-1.18, 0.06	n.s.
Parity	0.05	-0.38, 0.48	n.s.
BMI	0.04	-0.03, 0.11	n.s.
Current distress level	-0.14	-0.34, 0.06	n.s.
CpG 4	-0.02	-0.04, 0.01	n.s.
Maternal Age	0.01	-0.06, 0.09	n.s.
Medical history of depression	-0.70	-1.30, -0.10	n.s.
Pregnancy complications	-0.55	-1.17, 0.06	n.s.
Parity	0.05	-0.38, 0.48	n.s.
BMI	0.04	-0.03, 0.11	n.s.
Current distress level	-0.14	-0.34, 0.06	n.s.
Mean methylation level	-0.03	-0.06, 0.01	n.s.

Note: BMI=body mass index

Supplementary Table 3. Linear regression analyses between *NR3C1* CpG sites and mean methylation in relation to postpartum depressive symptoms adjusted for current distress level endorsed by the traumatic event.

**Predictors**	**B**	**CI (95%)**	**p**
Maternal Age	0.04	-0.11, 0.18	n.s.
Medical history of depression	2.45	1.32, 3.58	<0.001
Pregnancy complications	0.12	-1.05, 1.29	n.s.
Parity	-1.11	-1.93, 0.30	0.008
BMI	0.03	-0.10, 0.16	n.s.
Current distress level	0.46	0.08, 0.84	n.s.
CpG 1	0.16	0.00, 0.32	n.s.
Maternal Age	0.04	-0.11, 0.18	n.s.
Medical history of depression	2.72	1.58, 3.86	<0.001
Pregnancy complications	0.05	-1.10, 1.21	n.s.
Parity	-1.13	-1.93, -0.33	0.006
BMI	0.03	-0.10, 0.16	n.s.
Current distress level	0.46	0.08, 0.83	n.s.
CpG 2	0.32	0.10, 0.54	0.005
Maternal Age	0.04	-0.11, 0.18	n.s.
Medical history of depression	2.54	1.40, 3.67	<0.001
Pregnancy complications	0.08	-1.09, 1.25	n.s.
Parity	-1.16	-1.97, -0.35	0.005
BMI	0.03	-0.10, 0.16	n.s.
Current distress level	0.46	0.09, 0.84	n.s.
CpG 3	0.05	0.01, 0.10	n.s.
Maternal Age	0.03	-0.11, 0.18	n.s.
Medical history of depression	2.51	1.38, 3.64	<0.001
Pregnancy complications	0.11	-1.06, 1.27	n.s.
Parity	-1.16	-1.97, -0.35	0.005
BMI	0.03	-0.10, 0.16	n.s.
Current distress level	0.45	0.07, 0.83	n.s.
CpG 4	0.05	0.01, 0.09	n.s.
Maternal Age	0.04	-0.11, 0.18	n.s.
Medical history of depression	2.53	1.40, 3.67	<0.001
Pregnancy complications	0.09	-1.08, 1.25	n.s.
Parity	-1.15	-1.96, 0.34	0.006
BMI	0.03	-0.10, 0.16	n.s.
Current distress level	0.45	0.08, 0.83	n.s.
Mean methylation level	0.09	0.02, 0.16	n.s.

Note: BMI=body mass index

Supplementary Table 4. Linear regression analyses between *NR3C1* CpG sites and mean methylation in relation to resilience adjusted for total number of traumatic events

Predictors	B	CI (95%)	p
Maternal Age	0.01	-0.06, 0.09	n.s.
Medical history of depression	-0.70	-1.29, -0.10	n.s.
Pregnancy complications	-0.59	-1.21, 0.02	n.s.
Parity	0.04	-0.38, 0.47	n.s.
BMI	0.04	-0.03, 0.11	n.s.
Number of total events	-0.09	-0.23, 0.06	n.s.
CpG 1	-0.06	-0.15, 0.02	n.s.
Maternal Age	0.01	-0.06, 0.09	n.s.
Medical history of depression	-0.77	-1.37, -0.16	n.s.
Pregnancy complications	-0.59	-1.21, 0.03	n.s.
Parity	0.06	-0.37, 0.48	n.s.
BMI	0.04	-0.03, 0.11	n.s.
Number of total events	-0.09	-0.23, 0.06	n.s.
CpG 2	-0.08	-0.20, 0.03	n.s.
Maternal Age	0.01	-0.07, 0.09	n.s.
Medical history of depression	-0.73	-1.32, -0.13	n.s
Pregnancy complications	-0.59	-1.21, 0.03	n.s.
Parity	0.06	-0.36, 0.49	n.s.
BMI	0.04	-0.03, 0.11	n.s.
Number of total events	-0.09	-0.23, 0.06	n.s.
CpG 3	-0.02	-0.04, 0.01	n.s.
Maternal Age	-0.01	-0.06, 0.09	n.s.
Medical history of depression	-0.72	-1.32, -0.12	n.s.
Pregnancy complications	-0.59	-1.21, 0.02	n.s.
Parity	0.06	-0.37, 0.49	n.s.
BMI	0.04	-0.03, 0.11	n.s.
Number of total events	-0.08	-0.23, 0.06	n.s.
CpG 4	-0.02	-0.04, 0.00	n.s.
Maternal Age	0.01	-0.06, 0.09	n.s.
Medical history of depression	-0.73	-1.32, -0.13	n.s.
Pregnancy complications	-0.59	-1.20, 0.03	n.s.
Parity	0.06	-0.37, 0.49	n.s.
BMI	0.04	-0.03, 0.11	n.s.
Number of total events	-0.09	-0.23, 0.06	n.s.
Mean methylation level	-0.03	-0.07, 0.01	n.s.

Note: BMI=body mass index

Supplementary Table 5. Linear regression analyses between NR3C1 CpG sites and mean methylation in relation to postpartum depressive symptoms adjusted for total number of traumatic events

**Predictors**	**B**	**CI (95%)**	**p**
Maternal Age	0.40	-0.11, 0.19	n.s.
Medical history of depression	2.57	1.43, 3.71	<0.001
Pregnancy complications	0.24	-0.94, 1.42	n.s.
Parity	-1.17	-1.99, -0.34	0.006
BMI	0.03	-0.10, 0.17	n.s.
Number of total events	0.22	-0.05, 0.50	n.s.
CpG 1	0.18	0.03, 0.34	n.s.
Maternal Age	0.04	-0.10, 0.19	n.s.
Medical history of depression	2.86	1.71, 4.00	<0.001
Pregnancy complications	0.17	-0.99, 1.33	n.s.
Parity	-1.19	-2.00, -0.38	0.004
BMI	0.03	-0.10, 0.16	n.s.
Number of total events	0.21	-0.06, 0.49	n.s.
CpG 2	0.34	0.11, 0.56	0.003
Maternal Age	0.04	-0.11, 0.19	n.s.
Medical history of depression	2.66	1.52, 3.80	<.001
Pregnancy complications	0.20	-0.98, 1.38	n.s.
Parity	-1.23	-2.04, -0.41	0.004
BMI	0.03	-0.10, 0.17	n.s.
Number of total events	0.22	-0.06, 0.49	n.s.
CpG 3	0.06	0.01, 0.10	n.s.
Maternal Age	0.04	-0.11, 0.19	n.s.
Medical history of depression	2.63	1.50, 3.77	<0.001
Pregnancy complications	0.23	-0.95, 1.40	n.s.
Parity	-1.22	-2.04, -0.40	0.004
BMI	0.04	-0.10, 0.17	n.s.
Number of total events	0.21	-0.06, 0.49	n.s.
CpG 4	0.05	0.01, 0.09	n.s.
Maternal Age	0.04	-0.11, 0.19	n.s.
Medical history of depression	2.66	1.52, 3.80	<0.001
Pregnancy complications	0.21	-0.97, 1.38	n.s.
Parity	-1.21	-2.03, -0.40	0.004
BMI	0.03	-0.10, 0.17	n.s.
Number of total events	0.22	-0.06, 0.49	n.s.
Mean methylation level	0.09	0.02, 0.17	0.009

Note: BMI=body mass index

Supplementary Table 6. Linear regression analyses between NR3C1 CpG sites and mean methylation in relation to pregnancy depressive symptoms adjusted for total number of traumatic events

Predictors	B	CI (95%)	p
Maternal Age	0.01	-0.16, 0.19	n.s.
Medical history of depression	2.07	0.74, 3.39	0.003
Pregnancy complications	0.10	-1.28, 1.48	n.s.
Parity	0.20	-0.93, 0.99	n.s.
BMI	0.11	-0.06, 0.27	n.s.
Number of total events	0.30	-0.02, 0.60	n.s.
CpG 4	0.05	0.00, 0.09	n.s.

Note: BMI=body mass index

Supplementary Table 7. Linear regression analyses between NR3C1 CpG sites in relation to resilience adjusted for interpersonal traumatic events.

Predictors	B	CI (95%)	p
Maternal Age	0.01	-0.06, 0.09	n.s.
Medical history of depression	-0.69	-1.29, -0.10	n.s.
Pregnancy complications	-0.59	-1.20, 0.03	n.s.
Parity	0.05	-0.38, 0.47	n.s.
BMI	0.04	-0.03, 0.11	n.s.
Number of interpersonal events	-0.21	-0.52, 0.10	n.s.
CpG 1	-0.07	-0.15, 0.02	n.s.
Maternal Age	0.01	-0.06, 0.09	n.s.
Medical history of depression	-0.76	-1.37, -0.16	n.s.
Pregnancy complications	-0.58	-1.20, 0.03	n.s.
Parity	0.06	-0.37, 0.49	n.s.
BMI	0.04	-0.03, 0.11	n.s.
Number of interpersonal events	-0.19	-0.50, 0.11	n.s.
CpG 2	-0.08	-0.20, 0.04	n.s.
Maternal Age	0.01	-0.06, 0.09	n.s.
Medical history of depression	-0.72	-1.32, -0.13	n.s
Pregnancy complications	-0.58	-1.20, 0.03	n.s.
Parity	0.07	-0.36, 0.50	n.s.
BMI	0.04	-0.03, 0.11	n.s.
Number of interpersonal events	-0.20	-0.51, 0.11	n.s.
CpG 3	-0.02	-0.04, 0.01	n.s.
Maternal Age	0.01	-0.06, 0.09	n.s.
Medical history of depression	-0.72	-1.31, -0.12	n.s.
Pregnancy complications	-0.59	-1.20, 0.03	n.s.
Parity	0.06	-0.36, 0.49	n.s.
BMI	0.04	-0.03, 0.11	n.s.
Number of interpersonal events	-0.20	-0.50, 0.11	n.s.
CpG 4	-0.02	-0.04, 0.00	n.s.
Maternal Age	0.01	-0.06, 0.09	n.s.
Medical history of depression	-0.72	-1.32, -0.13	n.s.
Pregnancy complications	-0.58	-1.20, 0.03	n.s.
Parity	0.06	-0.36, 0.49	n.s.
BMI	0.04	-0.03, 0.11	n.s.
Number of interpersonal events	-0.20	-0.51, 0.11	n.s.
Mean methylation level	-0.03	-0.07, 0.01	n.s.

Note: BMI=body mass index

Supplementary Table 8. Linear regression analyses between NR3C1 CpG sites in relation to postpartum depressive symptoms adjusted for interpersonal traumatic events.

Predictors	B	CI (95%)	p
Maternal Age	0.04	-0.11, 0.19	n.s.
Medical history of depression	2.57	1.43, 3.71	<0.001
Pregnancy complications	0.23	-0.95, 1.41	n.s.
Parity	-1.17	-2.00, -0.35	0.006
BMI	0.03	-0.10, 0.17	n.s.
Number of interpersonal events	0.47	-0.12, 1.06	n.s.
CpG 1	0.19	0.03, 0.35	n.s.
Maternal Age	0.04	-0.10, 0.19	n.s.
Medical history of depression	2.87	1.73, 4.02	<0.001
Pregnancy complications	0.17	-1.00, 1.33	n.s.
Parity	-1.20	-2.01, -0.39	0.004
BMI	0.03	-0.10, 0.17	n.s.
Number of interpersonal events	0.41	-0.17, 0.99	n.s.
CpG 2	0.34	0.11, 0.56	0.003
Maternal Age	0.04	-0.11, 0.19	n.s.
Medical history of depression	2.68	1.53, 3.82	<0.001
Pregnancy complications	0.19	-0.99, 1.37	n.s.
Parity	-1.23	-2.05, -0.42	0.003
BMI	0.04	-0.10, 0.17	n.s.
Number of interpersonal events	0.44	-0.15, 1.03	n.s.
CpG 3	0.06	0.01, 0.10	n.s.
Maternal Age	0.04	-0.11, 0.19	n.s.
Medical history of depression	2.64	1.51, 3.78	<0.001
Pregnancy complications	0.22	-0.96, 1.39	n.s.
Parity	-1.23	-2.05, -0.41	0.004
BMI	0.04	-0.10, 0.17	n.s.
Number of interpersonal events	0.44	-0.15, 1.02	n.s.
CpG 4	0.05	0.01, 0.09	n.s
Maternal Age	0.04	-0.11, 0.19	n.s.
Medical history of depression	2.67	1.53, 3.81	n.s.
Pregnancy complications	0.20	-0.98, 1.37	n.s.
Parity	-1.22	-2.04, -0.40	<0.001
BMI	0.04	-0.10, 0.17	n.s.
Number of interpersonal events	0.44	-0.14, 1.02	n.s.
Mean methylation level	0.10	0.02, 0.17	0.008

Note: BMI=body mass index
